# Supplementary figures and images for: Metabolic Contributions of an Alphaproteobacterial Endosymbiont in the Apicomplexan Cardiosporidium cionae
Source: Front Microbiol. 2020 Dec 1;11:580719. doi: 10.3389/fmicb.2020.580719 (PMC7737231; doi:10.3389/fmicb.2020.580719)

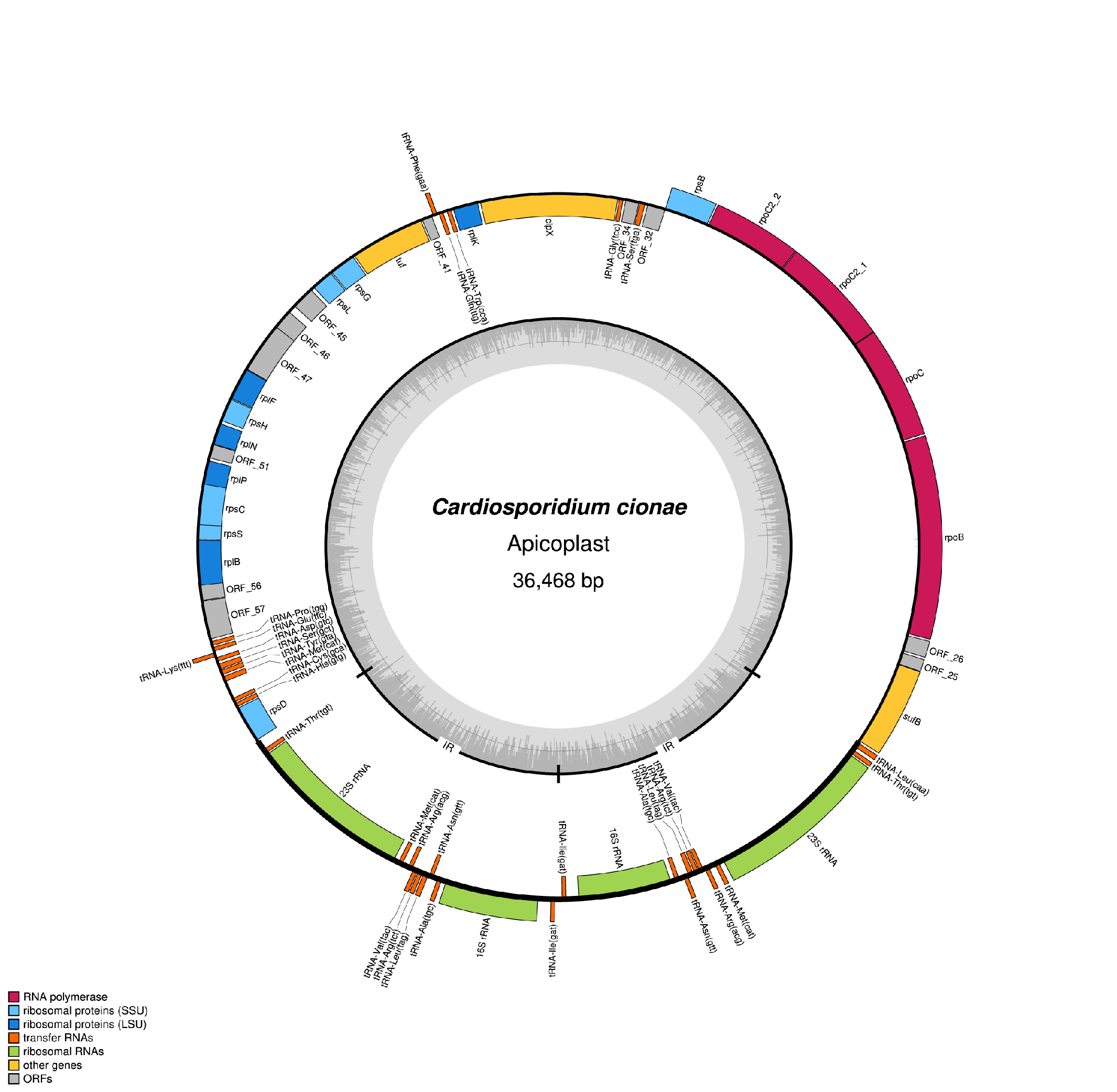

Supplement: Supplementary Figure 1 — The annotated, circularized Cardiosporidium cionae apicoplast. Two C. cionae apicoplasts were recovered that were 99.55% similar overall and contained identical gene organization, with the SNPs localized to the sufB gene. The C. cionae apicoplasts are similar in size, organization, and gene content to the Nephromyces apicoplasts. [file Image_1.JPEG]

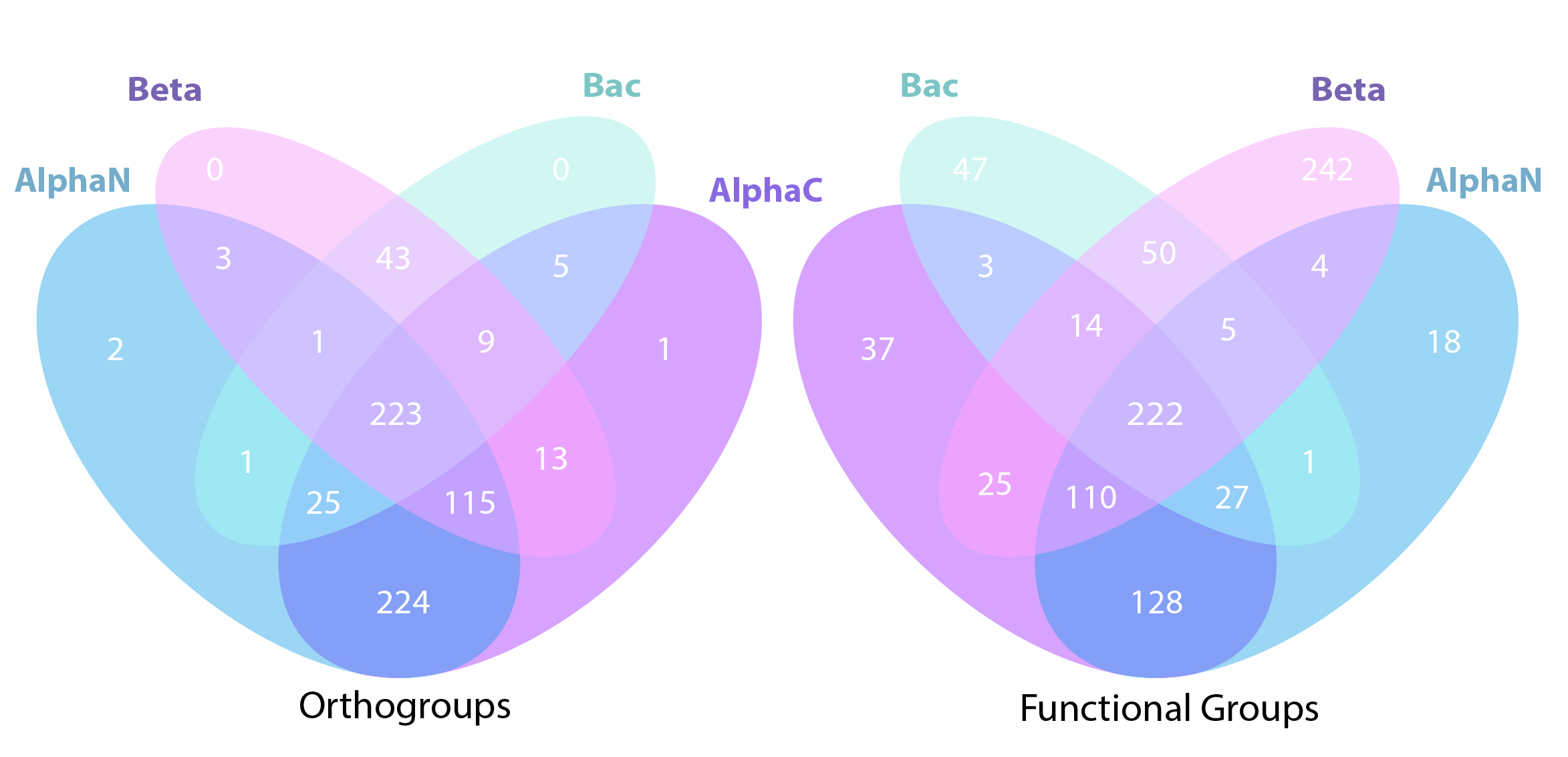

Supplement: Supplementary Figure 2 — Comparisons of the bacterial endosymbiont genomes in Cardiosporidium cionae (AlphaC) and Nephromyces (AlphaN, Beta, Bac). The left Venn diagram depicts orthologous groups predicted by OrthoFinder, while the right shows functional overlap predicted with KEGG. [file Image_2.JPEG]

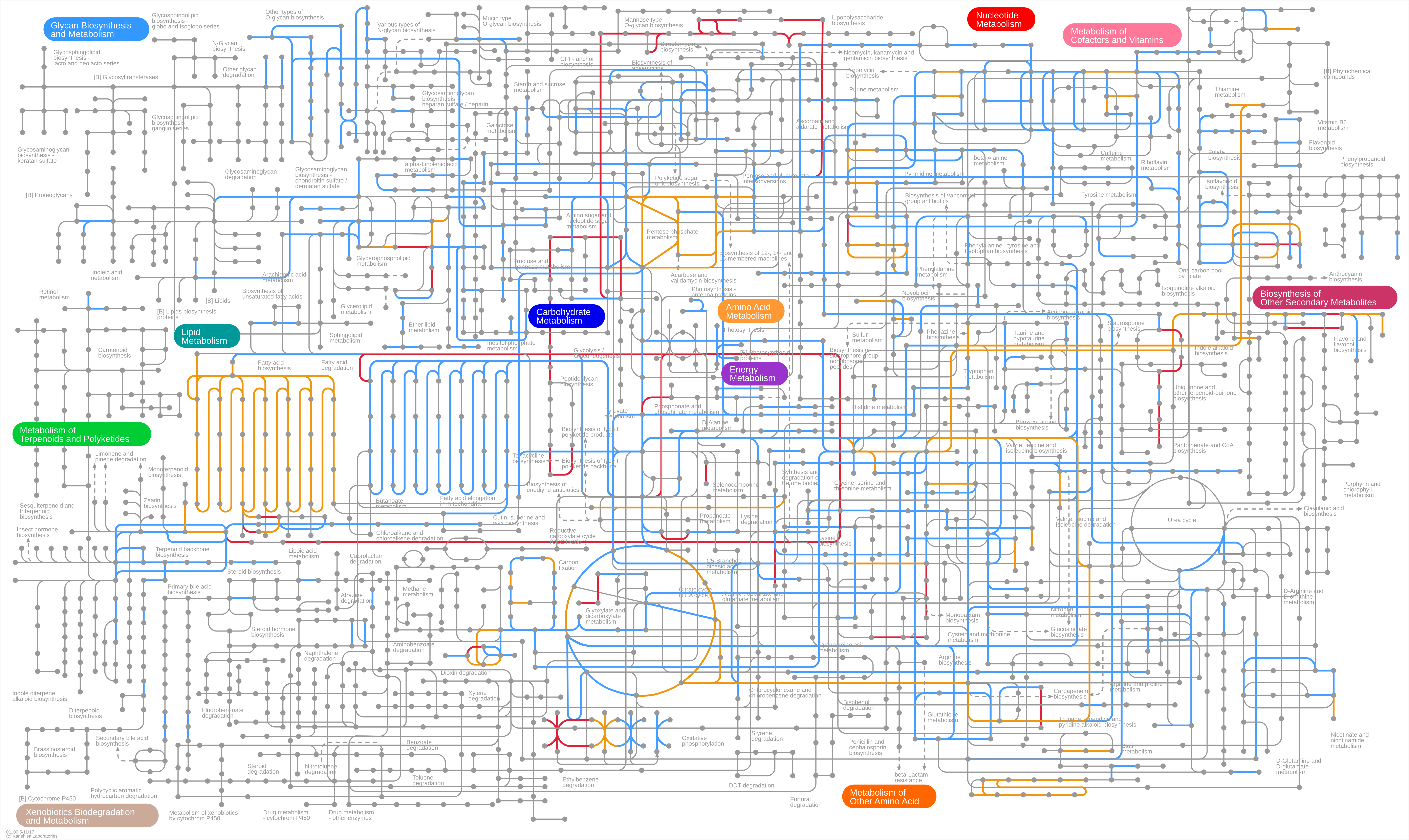

Supplement: Supplementary Figure 3 — Global metabolic map of the Cardiosporidium cionae genome and it’s α-proteobacterial endosymbiont, with pathways exclusive to C. cionae shown in blue, exclusive to the α-endosymbiont shown in red, and the overlapping metabolic pathways shown in yellow. This figure was generated with iPath 3 (Darzi et al., 2018). [file Image_3.PNG]
