# Supplementary material for: Metabolic Contributions of an Alphaproteobacterial Endosymbiont in the Apicomplexan Cardiosporidium cionae
Source: Front Microbiol. 2020 Dec 1;11:580719. doi: 10.3389/fmicb.2020.580719 (PMC7737231; doi:10.3389/fmicb.2020.580719)
Supplement: Supplementary Table 1 — Closely related reference genomes used for annotation of the Cardiosporidium cionaeα-endosymbiont, downloaded from GenBank, refseq. [file Table_1.docx]

Supplementary Table 1: Closely related reference genomes used for annotation of the *Cardiosporidium cionae* 𝝰-endosymbiont, downloaded from GenBank, refseq.

| ***Rickettsia* reference genomes** | **RefSeq Accession** |
| --- | --- |
| Rickettsia conorii str. Malish 7 | GCF_000007025.1 |
| Rickettsia felis str. Pedreira | GCF_000964665.1 |
| Rickettsia japonica YH | GCF_000283595.1 |
| Rickettsia massiliae MTU5 | GCF_000016625.1 |
| Rickettsia peacockii str. Rustic | GCF_000021525.1 |
| Rickettsia prowazekii str. Breinl | GCF_000367405.1 |
| Rickettsia typhi str. Wilmington | GCF_000008045.1 |
| Rickettsia bellii RML369-C | GCF_000012385.1 |
