# Supplementary material for: Metabolic Contributions of an Alphaproteobacterial Endosymbiont in the Apicomplexan Cardiosporidium cionae
Source: Front Microbiol. 2020 Dec 1;11:580719. doi: 10.3389/fmicb.2020.580719 (PMC7737231; doi:10.3389/fmicb.2020.580719)
Supplement: Supplementary Table 2 — Key functional genes annotations from the genome and transcriptome of Cardiosporidium cionae, corresponding to those seen in Figure 2, beginning with the top middle slice and moving clockwise. Annotations are based on functional predictions from KEGG and referenced by the corresponding K-Number. [file Table_2.docx]

Supplementary Table 2: Key functional genes annotations from the genome and transcriptome of *Cardiosporidium cionae,* corresponding to those seen in Figure 2, beginning with the top middle slice and moving clockwise. Annotations are based on functional predictions from KEGG and referenced by the corresponding K-Number.

| \| **Pathway/Step** \| **Description** \| **KNUM** \| **DNA** \| **RNA** \| \| --- \| --- \| --- \| --- \| --- \| \| **Glycolysis, glucose => pyruvate** \|  \|  \|  \|  \| \| **1** \| hexokinase \| K00844 \| x \| x \| \| **2** \| glucose-6-phosphate isomerase \| K01810 \| x \| x \| \| **3** \| 6-phosphofructokinase 1 \| K00805 \|  \|  \| \| **4** \| fructose-1,6-bisphosphate aldolase \| K01623 \| x \| x \| \| **5** \| triosephosphate isomerase (TIM) \| K01803 \| x \| x \| \| **6** \| glyceraldehyde 3-phosphate dehydrogenase \| K00134 \| x \| x \| \| **7** \| phosphoglycerate kinase \| K00927 \| x \| x \| \| **8** \| 2,3-bisphosphoglycerate-dependent phosphoglycerate mutase \| K01834 \| x \| x \| \| **9** \| enolase \| K01689 \| x \| x \| \| **10** \| pyruvate kinase \| K00873 \| x \| x \| \| **Gluconeogenesis, oxaloacetate => fructose-6P** \|  \|  \|  \|  \| \| **1** \| phosphoenolpyruvate carboxykinase (ATP) \| K01610 \| x \| x \| \| **2** \| enolase \| K01689 \| x \| x \| \| **3** \| 2,3-bisphosphoglycerate-dependent phosphoglycerate mutase \| K01834 \| x \| x \| \| **4** \| phosphoglycerate kinase \| K00927 \| x \| x \| \| **5** \| glyceraldehyde 3-phosphate dehydrogenase \| K00134 \| x \| x \| \| **6** \| triosephosphate isomerase (TIM) \| K01803 \| x \| x \| \| **7** \| fructose-bisphosphate aldolase, class I \| K01623 \| x \| x \| \| **8** \| fructose-1,6-bisphosphatase I + II \| K03841 \| x \| x \| \| **Pyruvate Oxidation, pyruvate => acetyl-CoA** \|  \|  \|  \|  \| \| **1** \| pyruvate dehydrogenase E1 component alpha subunit \| K00161 \| x \| x \| \| **2** \| pyruvate dehydrogenase E1 component beta subunit \| K00162 \| x \|  \| \| **3** \| pyruvate dehydrogenase E2 component (dihydrolipoamide acetyltransferase) \| K00627 \| x \| x \| \| **4** \| dihydrolipoamide dehydrogenase \| K00382 \|  \| x \| \| **5** \| dihydrolipoamide dehydrogenase-binding protein of pyruvate dehydrogenase complex \| K13997 \| x \|  \| \| **Citric Acid Cycle** \|  \|  \|  \|  \| \| **1** \| citrate synthase \| K01647 \| x \| x \| \| **2** \| aconitate hydratase \| K01681 \| x \| x \| \| **3** \| isocitrate dehydrogenase \| K00031 \| x \| x \| \| **4** \| 2-oxoglutarate dehydrogenase E1 component \| K00164 \| x \| x \| \| **5** \| 2-oxoglutarate dehydrogenase E2 component (dihydrolipoamide succinyltransferase) \| K00658 \| x \| x \| \| **6** \| pyruvate dehydrogenase complex subunit PDH-E3I \| K00382 \|  \| x \| \| **7** \| succinyl-CoA synthetase alpha subunit \| K01899 \| x \| x \| \| **8** \| succinyl-CoA synthetase beta subunit \| K01900 \| x \| x \| \| **9** \| succinate dehydrogenase (ubiquinone) flavoprotein subunit \| K00234 \| x \| x \| \| **10** \| succinate dehydrogenase (ubiquinone) iron-sulfur subunit \| K00235 \| x \| x \| \| **11** \| succinate dehydrogenase (ubiquinone) cytochrome b560 subunit \| K00236 \|  \|  \| \| **12** \| succinate dehydrogenase (ubiquinone) membrane anchor subunit \| K00237 \|  \|  \| \| **13** \| fumarate hydratase, class II \| K01679 \| x \|  \| \| **14** \| malate dehydrogenase \| K00024 \| x \| x \| \| **Pentose Phosphate Pathway** \|  \|  \|  \|  \| \| **1** \| glucose-6-phosphate 1-dehydrogenase \| K00036 \| x \| x \| \| **2** \| 6-phosphogluconolactonase \| K01057 \|  \|  \| \| **3** \| 6-phosphogluconate dehydrogenase \| K00033 \|  \| x \| \| **4** \| ribulose-phosphate 3-epimerase \| K01783 \| x \|  \| \| **5** \| ribose 5-phosphate isomerase A \| K01807 \| x \| x \| \| **6** \| transketolase \| K00615 \| x \| x \| \| **7** \| transaldolase \| K00616 \| x \| x \| \| **8** \| glucose-6-phosphate isomerase \| K01810 \| x \| x \| \| **Glyoxylate Cycle** \|  \|  \|  \|  \| \| **1** \| citrate synthase \| K01647 \| x \| x \| \| **2** \| aconitate hydratase \| K01681 \| x \| x \| \| **3** \| isocitrate lyase \| K01637 \|  \|  \| \| **4** \| malate synthase \| K01638 \|  \|  \| \| **5** \| malate dehydrogenase \| K00024 \| x \| x \| \| **Fatty Acid Biosynthesis (Initiation)** \|  \|  \|  \|  \| \| **1** \| acetyl-CoA carboxylase / biotin carboxylase 1 \| K11262 \| x \| x \| \| **2** \| [acyl-carrier-protein] S-malonyltransferase \| K00645 \| x \| x \| \| **3** \| 3-oxoacyl-[acyl-carrier-protein] synthase III \| K00648 \|  \| x \| \| **Fatty Acid Biosynthesis (Elongation)** \|  \|  \|  \|  \| \| **1** \| 3-oxoacyl-[acyl-carrier-protein] synthase II \| K09458 \| x \| x \| \| **2** \| 3-oxoacyl-[acyl-carrier protein] reductase \| K00059 \| x \|  \| \| **3** \| beta-hydroxyacyl-acyl carrier protein dehydratase (FABZ) \| K02372 \| x \| x \| \| **4** \| enoyl-[acyl-carrier protein] reductase I \| K00208 \| x \| x \| \| **Cholesterol Biosynthesis, squalene 2,3-epoxide => cholesterol** \|  \|  \|  \|  \| \| **1** \| lanosterol synthase \| K01852 \|  \|  \| \| **2** \| sterol 14alpha-demethylase \| K05917 \|  \|  \| \| **3** \| Delta14-sterol reductase \| K00222 \|  \|  \| \| **4** \| methylsterol monooxygenase \| K07750 \|  \| x \| \| **5** \| sterol-4alpha-carboxylate 3-dehydrogenase (decarboxylating) \| K07748 \|  \|  \| \| **6** \| 17beta-estradiol 17-dehydrogenase / 3beta-hydroxysteroid 3-dehydrogenase \| K13373 \|  \|  \| \| **7** \| Delta24-sterol reductase \| K09828 \|  \|  \| \| **8** \| cholestenol Delta-isomerase \| K01824 \|  \|  \| \| **9** \| C-5 sterol desaturase, putative \| K00227 \| x \|  \| \| **10** \| 7-dehydrocholesterol reductase, putative \| K00213 \| x \| x \| \| **Triaglycerol Biosynthesis** \|  \|  \|  \|  \| \| **1** \| glycerol-3-phosphate O-acyltransferase / dihydroxyacetone phosphate acyltransferase \| K13507 \| x \| x \| \| **2** \| glycerol-3-phosphate O-acyltransferase \| K00655 \|  \| x \| \| **2** \| 1-acyl-sn-glycerol-3-phosphate acyltransferase \| K22831 \| x \|  \| \| **3** \| 1-acylglycerol-3-phosphate O-acyltransferase \|  \|  \|  \| \| **4** \| diacylglycerol O-acyltransferase 1 \| K11155 \| x \| x \| \| **Ketone Body Biosynthesis, acetyl-CoA => acetoacetate/3-hydroxybutyrate/acetone** \|  \|  \|  \|  \| \| **1** \| acetoacetate decarboxylase \| K01574 \|  \|  \| \| **2** \| 3-hydroxybutyrate dehydrogenase \| K00019 \| x \|  \| \| **3** \| acetyl-CoA C-acetyltransferase \| K00626 \| x \| x \| \| **4** \| hydroxymethylglutaryl-CoA synthase \| K01641 \|  \|  \| \| **5** \| hydroxymethylglutaryl-CoA lyase \| K01640 \| x \|  \| \| **Beta Oxidation** \|  \|  \|  \|  \| \| **1** \| acyl-CoA oxidase \| K00232 \| x \| x \| \| **2** \| enoyl-CoA hydratase \| K07511 \| x \| x \| \| **3** \|  \|  \|  \|  \| \| **4** \| acetyl-CoA acyltransferase \| K07513 \| x \| x \| \| **IMP Biosynthesis** \|  \|  \|  \|  \| \| **1** \| amidophosphoribosyltransferase \| K00764 \|  \| x \| \| **2** \| phosphoribosylamine---glycine ligase \| K01945 \| x \|  \| \| **3** \| Trifunctional GART (fragment) \| K11787 \|  \| x \| \| **4** \| phosphoribosylformylglycinamidine synthase \| K01952 \|  \| x \| \| **5** \| Trifunctional GART (fragment) \| K11787 \|  \| x \| \| **6** \| phosphoribosylaminoimidazole-succinocarboxamide synthase \| K01923 \| x \| x \| \| **7** \| adenylosuccinate lyase \| K01756 \| x \|  \| \| **8** \| phosphoribosylaminoimidazolecarboxamide formyltransferase \| K00602 \| x \|  \| \| **Guanine Biosynthesis** \|  \|  \|  \|  \| \| **1** \| IMP dehydrogenase \| K00088 \| x \| x \| \| **2** \| GMP synthase (glutamine-hydrolysing) \| K01951 \| x \| x \| \| **3** \| guanylate kinase \| K00942 \| x \| x \| \| **4** \| pyruvate kinase \| K00873 \| x \| x \| \| **4** \| nucleoside-diphosphate kinase \| K00940 \| x \|  \| \| **Adenine Biosynthesis** \|  \|  \|  \|  \| \| **1** \| adenylosuccinate lyase \| K00939 \| x \|  \| \| **2** \| adenylosuccinate synthase \| K01756 \| x \| x \| \| **3** \| adenylate kinase \| K01939 \| x \| x \| \| **4** \| nucleoside-diphosphate kinase \| K00940 \| x \|  \| \| **4** \| pyruvate kinase \| K00873 \| x \| x \| \| **Pyrimidine Biosynthesis (ribonucleotide)** \|  \|  \|  \|  \| \| **1** \| UMP-CMP kinase \| K13800 \| x \| x \| \| **2** \| nucleoside-diphosphate kinase \| K00940 \| x \|  \| \| **3** \| CTP synthase \| K01937 \| x \| x \| \| **Pyrimidine Biosynthesis (deoxyribonucleotide)** \|  \|  \|  \|  \| \| **1** \| ribonucleoside-diphosphate reductase subunit M1 \| K10807 \| x \| x \| \| **2** \| ribonucleoside-diphosphate reductase subunit M2 \| K10808 \| x \| x \| \| **3** \| nucleoside-diphosphate kinase \| K00940 \| x \|  \| \| **4** \|  \|  \|  \|  \| \| **5** \| dCTP deaminase \| K01494 \|  \|  \| \| **6** \| dUTP pyrophosphatase \| K01520 \| x \| x \| \| **7** \| dihydrofolate reductase / thymidylate synthase \| K13998 \|  \| x \| \| **8** \| thymidylate kinase \| K00943 \| x \| x \| \| **9** \| nucleoside-diphosphate kinase \| K00940 \| x \|  \| \| **Uridine Biosynthesis** \|  \|  \|  \|  \| \| **1** \| aspartate carbamoyltransferase \|  \| x \|  \| \| **1** \| carbamoyl-phosphate synthase \| K11541 \|  \| x \| \| **1** \| dihydroorotase \| K01465 \| x \| x \| \| **2** \| dihydroorotate dehydrogenase \| K00254 \| x \| x \| \| **3** \| orotate phosphoribosyltransferase \| K00762 \| x \|  \| \| **4** \| orotidine-5'-phosphate decarboxylase \| K01591 \| x \| x \| \| **Alanine, pyruvate => alanine (L)** \|  \|  \|  \|  \| \| **1** \| alanine dehydrogenase \| K00259 \| x \|  \| \| **Arginine** \| N/A \|  \|  \|  \| \| **Asparagine, pyruvate => asparagine** \|  \|  \|  \|  \| \| **1** \| pyruvate carboxylase \| K01958 \| x \|  \| \| **2** \| aspartate aminotransferase, cytoplasmic \| K14454 \| x \| x \| \| **2** \| aspartate aminotransferase, mitochondrial \| K14455 \| x \| x \| \| **3** \| asparagine synthase \| K01953 \| x \| x \| \| **Aspartic Acid, pyruvate => aspartate** \|  \|  \|  \|  \| \| **1** \| pyruvate carboxylase \| K01958 \| x \|  \| \| **2** \| aspartate aminotransferase, cytoplasmic \| K14454 \| x \| x \| \| **2** \| aspartate aminotransferase, mitochondrial \| K14455 \| x \| x \| \| **Cysteine biosynthesis, homocysteine + serine => cysteine** \|  \|  \|  \|  \| \| **1** \| cystathionine beta-synthase \| K01697 \| x \|  \| \| **2** \| cystathionine gamma-lyase \| K01758 \|  \|  \| \| **Glutamic Acid** \|  \|  \|  \|  \| \| **1** \| pyruvate carboxylase \| K01958 \| x \|  \| \| **2** \| citrate synthase \| K01647 \| x \| x \| \| **3** \| aconitate hydratase \| K01681 \| x \| x \| \| **4** \| isocitrate dehydrogenase \| K00031 \| x \| x \| \| **5** \| aspartate aminotransferase, cytoplasmic \| K14454 \| x \| x \| \| **Glutamine, pyruvate => glutamine** \|  \|  \|  \|  \| \| **1** \| pyruvate carboxylase \| K01958 \| x \| x \| \| **2** \| citrate synthase \| K01647 \| x \| x \| \| **3** \| aconitate hydratase \| K01681 \| x \| x \| \| **4** \| isocitrate dehydrogenase \| K00031 \| x \| x \| \| **5** \| aspartate aminotransferase, cytoplasmic \| K14454 \| x \| x \| \| **6** \| glutamine synthetase \| K01915 \| x \| x \| \| **Glycine, glyoxylate => glycine** \|  \|  \|  \|  \| \| **1** \| alanine-glyoxylate transaminase / serine-glyoxylate transaminase / serine-pyruvate transaminase \| K00830 \|  \| x \| \| **Glycine, 3P-D-Glycerate => glycine** \|  \|  \|  \|  \| \| **1** \| D-3-phosphoglycerate dehydrogenase / 2-oxoglutarate reductase \| K00058 \| x \| x \| \| **2** \| phosphoserine aminotransferase \| K00831 \| x \|  \| \| **3** \| phosphoserine phosphatase \| K01079 \| x \| x \| \| **4** \| glycine hydroxymethyltransferase \| K00600 \|  \|  \| \| **Histidine** \| N/A \|  \|  \|  \| \| **Isoleucine** \| N/A \|  \|  \|  \| \| **Leucine** \| N/A \|  \|  \|  \| \| **Lysine** \| N/A \|  \|  \|  \| \| **Methionine biosynthesis, apartate => homoserine => methionine** \|  \|  \|  \|  \| \| **1** \| bifunctional aspartokinase / homoserine dehydrogenase 1 \| K12524 \| x \| x \| \| **2** \| aspartate-semialdehyde dehydrogenase \| K00133 \| x \| x \| \| **3** \| bifunctional aspartokinase / homoserine dehydrogenase 1 \| K12524 \| x \| x \| \| **4** \|  \|  \|  \|  \| \| **5** \| cystathionine gamma-synthase \| K01739 \|  \|  \| \| **6** \|  \|  \|  \|  \| \| **7** \| 5-methyltetrahydropteroyltriglutamate--homocysteine methyltransferase \| K00549 \| x \| x \| \| **Phenylalanine, phosphoenol-pyruvate => phenylalanine** \|  \|  \|  \|  \| \| **1** \| 3-deoxy-7-phosphoheptulonate synthase \| K03856 \|  \|  \| \| **2** \| 3-dehydroquinate synthase \| K01735 \|  \| x \| \| **3** \| pentafunctional AROM polypeptide \| K13830 \|  \| x \| \| **4** \| pentafunctional AROM polypeptide \| K13830 \|  \| x \| \| **5** \|  \|  \|  \|  \| \| **6** \|  \|  \|  \|  \| \| **7** \| pentafunctional AROM polypeptide \| K13830 \|  \| x \| \| **8** \| pentafunctional AROM polypeptide \| K13830 \|  \| x \| \| **9** \| chorismate synthase \| K01736 \| x \|  \| \| **10** \|  \|  \|  \|  \| \| **11** \|  \|  \|  \|  \| \| **12** \|  \|  \|  \|  \| \| **13** \| aspartate aminotransferase, cytoplasmic \| K14454 \| x \| x \| \| **14** \|  \|  \|  \|  \| \| **Proline** \| N/A \|  \|  \|  \| \| **Serine biosynthesis, glycerate-3P => serine** \|  \|  \|  \|  \| \| **1** \| D-3-phosphoglycerate dehydrogenase / 2-oxoglutarate reductase \| K00058 \| x \| x \| \| **2** \| phosphoserine aminotransferase \| K00831 \| x \|  \| \| **3** \| phosphoserine phosphatase \| K01079 \| x \| x \| \| **Threonine biosynthesis, aspartate => homoserine => threonine** \|  \|  \|  \|  \| \| **1** \| bifunctional aspartokinase / homoserine dehydrogenase 1 \| K12524 \| x \| x \| \| **2** \| aspartate-semialdehyde dehydrogenase \| K00133 \| x \| x \| \| **3** \| bifunctional aspartokinase / homoserine dehydrogenase 1 \| K12524 \| x \| x \| \| **4** \|  \|  \|  \|  \| \| **5** \| threonine synthase \| K01733 \| x \| x \| \| **Tryptophan** \| N/A \|  \|  \|  \| \| **Tyrosine, phenylalanine => tyrosine** \|  \|  \|  \|  \| \| **1** \| phenylalanine-4-hydroxylase, putative \| K00500 \| x \| x \| \| **Valine** \| no genes present \|  \|  \|  \| |
| --- | --- | --- | --- | --- | --- | --- | --- | --- | --- | --- | --- | --- | --- | --- | --- | --- | --- | --- | --- | --- | --- | --- | --- | --- | --- | --- | --- | --- | --- | --- | --- | --- | --- | --- | --- | --- | --- | --- | --- | --- | --- | --- | --- | --- | --- | --- | --- | --- | --- | --- | --- | --- | --- | --- | --- | --- | --- | --- | --- | --- | --- | --- | --- | --- | --- | --- | --- | --- | --- | --- | --- | --- | --- | --- | --- | --- | --- | --- | --- | --- | --- | --- | --- | --- | --- | --- | --- | --- | --- | --- | --- | --- | --- | --- | --- | --- | --- | --- | --- | --- | --- | --- | --- | --- | --- | --- | --- | --- | --- | --- | --- | --- | --- | --- | --- | --- | --- | --- | --- | --- | --- | --- | --- | --- | --- | --- | --- | --- | --- | --- | --- | --- | --- | --- | --- | --- | --- | --- | --- | --- | --- | --- | --- | --- | --- | --- | --- | --- | --- | --- | --- | --- | --- | --- | --- | --- | --- | --- | --- | --- | --- | --- | --- | --- | --- | --- | --- | --- | --- | --- | --- | --- | --- | --- | --- | --- | --- | --- | --- | --- | --- | --- | --- | --- | --- | --- | --- | --- | --- | --- | --- | --- | --- | --- | --- | --- | --- | --- | --- | --- | --- | --- | --- | --- | --- | --- | --- | --- | --- | --- | --- | --- | --- | --- | --- | --- | --- | --- | --- | --- | --- | --- | --- | --- | --- | --- | --- | --- | --- | --- | --- | --- | --- | --- | --- | --- | --- | --- | --- | --- | --- | --- | --- | --- | --- | --- | --- | --- | --- | --- | --- | --- | --- | --- | --- | --- | --- | --- | --- | --- | --- | --- | --- | --- | --- | --- | --- | --- | --- | --- | --- | --- | --- | --- | --- | --- | --- | --- | --- | --- | --- | --- | --- | --- | --- | --- | --- | --- | --- | --- | --- | --- | --- | --- | --- | --- | --- | --- | --- | --- | --- | --- | --- | --- | --- | --- | --- | --- | --- | --- | --- | --- | --- | --- | --- | --- | --- | --- | --- | --- | --- | --- | --- | --- | --- | --- | --- | --- | --- | --- | --- | --- | --- | --- | --- | --- | --- | --- | --- | --- | --- | --- | --- | --- | --- | --- | --- | --- | --- | --- | --- | --- | --- | --- | --- | --- | --- | --- | --- | --- | --- | --- | --- | --- | --- | --- | --- | --- | --- | --- | --- | --- | --- | --- | --- | --- | --- | --- | --- | --- | --- | --- | --- | --- | --- | --- | --- | --- | --- | --- | --- | --- | --- | --- | --- | --- | --- | --- | --- | --- | --- | --- | --- | --- | --- | --- | --- | --- | --- | --- | --- | --- | --- | --- | --- | --- | --- | --- | --- | --- | --- | --- | --- | --- | --- | --- | --- | --- | --- | --- | --- | --- | --- | --- | --- | --- | --- | --- | --- | --- | --- | --- | --- | --- | --- | --- | --- | --- | --- | --- | --- | --- | --- | --- | --- | --- | --- | --- | --- | --- | --- | --- | --- | --- | --- | --- | --- | --- | --- | --- | --- | --- | --- | --- | --- | --- | --- | --- | --- | --- | --- | --- | --- | --- | --- | --- | --- | --- | --- | --- | --- | --- | --- | --- | --- | --- | --- | --- | --- | --- | --- | --- | --- | --- | --- | --- | --- | --- | --- | --- | --- | --- | --- | --- | --- | --- | --- | --- | --- | --- | --- | --- | --- | --- | --- | --- | --- | --- | --- | --- | --- | --- | --- | --- | --- | --- | --- | --- | --- | --- | --- | --- | --- | --- | --- | --- | --- | --- | --- | --- | --- | --- | --- | --- | --- | --- | --- | --- | --- | --- | --- | --- | --- | --- | --- | --- | --- | --- | --- | --- | --- | --- | --- | --- | --- | --- | --- | --- | --- | --- | --- | --- | --- | --- | --- | --- | --- | --- | --- | --- | --- | --- | --- | --- | --- | --- | --- | --- | --- | --- | --- | --- | --- | --- | --- | --- | --- | --- | --- | --- | --- | --- | --- | --- | --- | --- | --- | --- | --- | --- | --- | --- | --- | --- | --- | --- | --- | --- | --- | --- | --- | --- | --- | --- | --- | --- | --- | --- | --- | --- | --- | --- | --- | --- | --- | --- | --- | --- | --- | --- | --- | --- | --- | --- | --- | --- | --- | --- | --- | --- | --- | --- | --- | --- | --- | --- | --- | --- | --- | --- | --- | --- | --- | --- | --- | --- | --- | --- | --- | --- | --- | --- | --- | --- | --- | --- | --- | --- | --- | --- | --- | --- | --- | --- | --- | --- | --- | --- | --- | --- | --- | --- | --- | --- | --- | --- | --- | --- | --- | --- | --- | --- | --- | --- | --- | --- | --- | --- | --- | --- | --- | --- | --- | --- | --- | --- | --- | --- | --- | --- | --- | --- | --- | --- | --- | --- | --- | --- | --- | --- | --- | --- | --- | --- | --- | --- | --- | --- | --- | --- | --- | --- | --- | --- | --- | --- | --- | --- | --- | --- | --- | --- | --- | --- | --- | --- | --- | --- | --- | --- | --- | --- | --- | --- | --- | --- | --- | --- | --- | --- | --- | --- | --- | --- | --- | --- | --- | --- | --- | --- | --- | --- | --- | --- | --- | --- | --- | --- | --- | --- | --- | --- | --- | --- | --- | --- | --- | --- | --- | --- | --- | --- | --- | --- | --- | --- | --- | --- | --- | --- | --- | --- | --- | --- | --- | --- | --- | --- | --- | --- | --- | --- | --- | --- | --- | --- | --- | --- | --- | --- | --- | --- | --- | --- | --- | --- | --- | --- | --- | --- | --- | --- | --- | --- | --- | --- | --- | --- | --- | --- | --- | --- | --- | --- | --- | --- | --- | --- | --- | --- | --- | --- | --- | --- | --- | --- | --- | --- | --- | --- | --- | --- | --- | --- | --- | --- | --- | --- | --- | --- | --- | --- | --- | --- | --- | --- | --- | --- | --- | --- | --- | --- | --- | --- | --- | --- | --- | --- | --- | --- | --- | --- | --- | --- | --- | --- | --- | --- | --- | --- | --- | --- | --- | --- | --- | --- | --- | --- | --- | --- | --- | --- | --- | --- | --- | --- | --- | --- | --- | --- | --- | --- | --- | --- | --- | --- | --- | --- | --- | --- | --- | --- | --- | --- | --- | --- | --- | --- | --- | --- | --- | --- | --- | --- | --- | --- | --- | --- | --- | --- | --- | --- | --- | --- | --- | --- | --- | --- | --- | --- | --- | --- | --- | --- | --- | --- | --- | --- | --- | --- | --- | --- | --- | --- | --- | --- | --- | --- | --- | --- | --- | --- | --- | --- | --- | --- | --- | --- | --- | --- | --- | --- | --- | --- | --- | --- | --- | --- | --- | --- | --- | --- | --- | --- | --- | --- | --- | --- | --- | --- | --- | --- | --- | --- | --- | --- | --- | --- | --- | --- | --- | --- | --- | --- | --- | --- | --- | --- | --- | --- | --- | --- | --- | --- | --- | --- | --- | --- | --- | --- | --- | --- | --- | --- | --- |
